# CD200R Combined Neutrophil-Lymphocyte Ratio Predict 90-Day Mortality in HBV-Related Acute-On-Chronic Liver Failure

**DOI:** 10.3389/fmed.2021.762296

**Published:** 2021-12-06

**Authors:** Yuxin Li, Yaxian Kong, Ke Shi, Yunyi Huang, Qun Zhang, Bingbing Zhu, Hui Zeng, Xianbo Wang

**Affiliations:** ^1^Center of Integrative Medicine, Beijing Ditan Hospital, Capital Medical University, Beijing, China; ^2^Beijing Key Laboratory of Emerging Infectious Diseases, Institute of Infectious Diseases, Beijing Ditan Hospital, Capital Medical University, Beijing, China; ^3^Biomedical Innovation Center, Beijing Shijitan Hospital, Capital Medical University, Beijing, China

**Keywords:** ACLF, CD200R, HBV, prognosis, T cell, NLR

## Abstract

**Background:** Survival of acute-on-chronic liver failure (ACLF) cannot be properly predicted based on clinical characteristics.

**Aims:** This study aimed to develop a predictive model to evaluating the prognosis for hepatitis B virus-related ACLF (HBV-ACLF) based on specific laboratory and immune indicators.

**Methods:** Baseline laboratory results were obtained and immune indicators were detected by flow cytometry. A predictive model, which estimates the prognosis at 90-day follow-up, was developed using data from a prospective study on 45 patients hospitalized of HBV-ACLF from June 2016 to April 2018 at the Beijing Ditan Hospital, Capital Medical University. The prognostic values of the predictive factors were determined by the area under the receiver operating characteristic (AUROC) curves.

**Results:** Six factors exhibited statistical differences between the survival and non-survival groups: proportions of CD4^+^T_N_, CD4^+^T_EM_, CD8^+^T_N_, CD8^+^T_EM_, CD200R^+^CD4^+^T cells and neutrophil-lymphocyte ratio (NLR). CD200R combined with the NLR had an AUROC of 0.916, which was significantly higher than the AUROC values of CD200R^+^CD4^+^T cells (0.868), NLR (0.761), model for end-stage liver disease (MELD) (0.840), MELD-Na (0.870), Child-Turcotte-Pugh (CTP) (0.580), or chronic liver failure-consortium ACLF (CLIF-C ACLF) score(0.840). At the cut-off point of−3.87, matching the maximum Youden index determined by ROC analysis, the positive predictive and negative predictive values for the mortality were 0.86 and 0.97, respectively.

**Conclusions:** The 90-day prediction model based on baseline levels of CD200R^+^CD4^+^T cells and NLR offers potential predictive value for the mortality of HBV-ACLF.

## Introduction

Acute-on-chronic liver failure (ACLF) is an acute deterioration of pre-existing chronic liver diseases, manifesting as jaundice, coagulopathy and complicated within 4 weeks by ascites and/or hepatic encephalopathy, ultimately resulting in high short-term mortality (1–3). In particular, hepatitis B virus (HBV) infections account for most cases in the Asia-Pacific region because of their high prevalence (1).

In patients with chronic hepatitis B (CHB), persistent exposure to antigens often leads to depressed T cell function, characterized by the multiple expression of coinhibitory molecules (4–6). The upregulation of inhibitory receptors, such as programmed cell death protein-1 (PD-1), cytotoxic T-lymphocyte associated protein-4 (CTLA-4), and 2B4 (CD244) (7–9), has been confirmed to be related to the dysfunction of HBV-specific CD8^+^ T cells in chronic infection. Hepatitis B virus-related ACLF (HBV-ACLF) is a more complicated disease with excessive inflammation and immune dysfunction. Multiple factors, particularly host immunity, are involved in the pathogenesis of HBV-ACLF (10–12). T cell dysfunction and increased expression of coinhibitory molecules are also involved in the pathogenesis of HBV-ACLF.

CD200 is a cell surface glycoprotein that functions by engaging the CD200 receptor on cells of the myeloid and lymphoid lineages to transmit signals affecting responses in multiple physiological systems (13, 14). CD200 expression has been reported to affect cancer growth, autoimmune and allergic disorders, infection, transplantation, bone development and homeostasis, and reproductive biology (15–17). However, its role in the pathogenesis of HBV-ACLF remains to be explored. Neutrophil-lymphocyte ratio (NLR) has been confirmed as a potential short-term prognostic indicator for patients with HBV-ACLF (18). In this study, we investigated the correlation between CD200R along with NLR and the prognosis of HBV-ACLF and whether CD200R combined NLR could be used to predict prognosis. These findings may contribute to a better understanding of the pathogenesis of HBV-ACLF, which could be helpful in clinical decision-making and the development of novel therapeutic methods.

## Patients and Methods

### Patients

A total of 45 patients with HBV-ACLF and 169 patients with CHB were enrolled between June 2016 and April 2018 at the Beijing Ditan Hospital, Capital Medical University. The inclusion criteria for CHB was set according to the Chinese guideline of prevention and treatment for CHB (2015 version) (19): (1) 18 years or older, (2) HBsAg positive status for at least 6 months. The inclusion criteria for HBV-ACLF patients were set according to the consensus recommendations of the Asian Pacific Association for the Study of the Liver (APASL) for ACLF (2014) (20): (1) age 18 years or older, (2) HBsAg positive status for at least 6 months, (3) serum bilirubin ≥ 5 mg/dL (85 μmol/L) and coagulopathy (international normalized ratio (INR) ≥ 1.5 or prothrombin activity (PTA) <40%) complicated within 4 weeks by clinical ascites and/or encephalopathy. The key exclusion criteria included pregnancy, mental illness, decompensated liver cirrhosis, hepatocellular carcinoma, liver transplants, immune regulatory treatment within 6 months, co-infection with hepatitis A, C, D, E, or liver diseases caused by other reasons. In addition, patients with CHB who meet the criteria of serum bilirubin ≥ 5 mg/dL (85 μmol/L) and coagulopathy (INR ≥ 1.5 or PTA <40%) was excluded from CHB group. Patients with HBV-ACLF were followed up for 90 days. Fifty healthy control (HC) subjects were also recruited for the study. The present study was approved by the Ethics Committee of the Beijing Ditan Hospital, Capital Medical University, China. Written informed consent was obtained from all the patients before their participation.

### Isolation of Peripheral Blood Mononuclear Cells (PBMCs)

Peripheral blood samples were collected from all the subjects. PBMCs were isolated by Ficoll-Paque PLUS (GE Healthcare Biosciences AB, Uppsala, Sweden) density gradient centrifugation.

### Cell Surface Staining

PBMCs were incubated with directly conjugated antibodies for 30 min at 4°C. The cells were washed before flow cytometric analysis. The antibodies used were anti-human CD3-BV786, CD4-APC-Fire750, CD8-BV510, CD45RA-AF700 (Becton, Dickinson, and Company [BD], Franklin Lakes, NJ, USA), and CD200R APC and CCR7-BV421 (BioLegend, San Diego, CA, USA). Additionally, 7-AAD (BD, Franklin Lakes, NJ, USA) was used to exclude non-viable cells.

### Multiparameter Flow Cytometry

Cells stained with fluorescent antibodies were acquired with an LSR Fortessa flow cytometer (BD Biosciences) and analyzed with FlowJo software (Tree Star, Ashland, OR, USA).

### Statistical Analysis

Statistical analysis was performed using GraphPad 6 (GraphPad Software, La Jolla, CA, USA) or SPSS 23.0 (IBM Corporation, New York, NY, USA). Clinical and demographic characteristics were summarized as the mean ± standard deviation, median and 25th and 75th percentiles for continuous variables, and frequency and percentage for categorical variables. The Kolmogorov–Smirnov test was used to assess the normality of the sample data distribution. Fisher's exact test or χ^2^ test was used to compare the categorical variables. A one-way ANOVA test or Kruskal–Wallis test was performed to compare two more independent samples. The accuracy of prognosis was evaluated using the receiver operating characteristic (ROC) curve, and the area under the ROC (AUROC) curve was calculated. For all analyses, a *p* value of < 0.05 was considered statistically significant.

## Results

### Patient Characteristics and Baselines

The demographics and characteristics of the subjects are shown in Table 1. The HBV-ACLF group displayed expected differences in liver function and coagulation function parameters compared with the CHB and HC groups. Higher levels of alanine aminotransferase (ALT), aspartate aminotransferase (AST), total bilirubin (TBIL), NLR and INR were observed in patients with HBV-ACLF. Notably, the increase in NLR was mainly caused by a decrease in lymphocyte count. Additionally, PTA and albumin levels were significantly lower (*p* < 0.001) than those in patients with CHB.

**Table 1:** Characteristics of the subjects included in the study.

**Variables**	**HC**	**CHB**	**ACLF**	***p*-value**
	***n* = 50**	***n* = 169**	***n* = 45**	
**Demographics**				
Age (year)	34.5 (29.0,43.0)	33.0 (28.0,42.0)	44.5 ± 12.8	<0.001
Male/Female,%	27/23	99/70	36/9	0.040
**Laboratory data**				
WBC (10^9^/L)	6.1 ± 12	5.4 ± 1.3	4.9 (4.0,6.7)	0.004
NC (10^9^/L)	3.6 ± 1.0	3.0 ± 1.0	3.0 (1.9,4.2)	0.006
LC (10^9^/L)	2.0 ± 0.5	1.8 (1.5,2.2)	1.4 ± 0.6	<0.001
NLR	1.9 ± 0.7	1.6 (1.2,2.0)	2.4 (1.6,3.6)	<0.001
ALT (U/L)	13.5 (10.0,25)	31.1 (23.0,200.6)	355.5 (130.8,832.9)	<0.001
AST (U/L)	17.1 (15.3,21.4)	24.0 (18.9,94.7)	217.9 (94.9,453.0)	<0.001
ALB (g/L)	47.9 (46.7,49.9)	47.0 (42.9,49.1)	32.9 ± 4.8	<0.001
GLO (g/L)	22.3 (21.1,25.0)	26.7 (24.2,29.4)	27.7 ± 6.7	<0.001
TBIL (μmoI/L)	11.6 (9.4,12.9)	13.8 (10.3,21.2)	232.7 (148.9,325.8)	<0.001
PTA (%)	/	94.3 ± 13.2	35.9 ± 10.5	<0.001
INR	/	1.1 (1.0,1.1)	2.1 (1.8,2.5)	<0.001
logHBVDNA log (IU/mL)	/	6.1 (3.4,8.1)	5.2 ± 1.7	0.160

*HC, healthy control; CHB, chronic hepatitis B; ACLF, acute-on-chronic liver failure; WBC, white blood cell; LC, lymphocyte count; NC, neutrophil count; NLR, neutrophil-lymphocytes ratio; ALT, alanine aminotransferase; AST, aspartate aminotransferase; ALB, albumin; GLO, globulin; TBIL, total bilirubin; PTA, prothrombin activity; INR, international normalized ratio; p values were calculated by χ^2^ for categorical variables and Kruskal-Wallis test or unpaired t test for continuous variables*.

In total, 41 patients were included for the analysis, 4 out of 45 patients were excluded for liver transplantation. The differences between the survival and non-survival groups are shown in Table 2. Higher levels of neutrophil count and INR and lower level of PTA were observed in the non-survival group, indicating that infection and worse coagulation function may contribute to the adverse outcomes.

**Table 2:** Characteristics of the survival and non-survival groups in patients with HBV-ACLF.

**Variables**	**Survival**	**Non-survival**	***p*-value**
	***n* = 34**	***n* = 7**	
**Demographics**			
Age (year)	44.0 (30.8,58.5)	47.0 (46.0,50.0)	0.859
Male/Female, %	25/9	7/0	0.315
**Laboratory data**			
WBC (10^9^/L)	4.7 (3.9,6.5)	5.4 (5.2,13.1)	0.079
NC (10^9^/L)	3.1 ± 1.3	6.4 ± 4.5	<0.001
LC (10^9^/L)	1.4 ± 0.6	1.2 ± 0.4	0.352
NLR	2.3 (1.4,2.7)	5.2 ± 3.3	0.031
PLT (10^9^/L)	79.0 (61.6,109.4)	125.7 ± 63.2	0.077
ALT (U/L)	344.2 (127.5,784.5)	739.6 ± 839.1	0.905
AST (U/L)	217.6 (87.2,472.4)	364.7 ± 276.4	0.552
ALB (g/L)	32.6 ± 4.6	33.2 ± 4.8	0.748
GLO (g/L)	28.0 ± 6.8	25.0 ± 7.5	0.312
TBIL (μmoI/L)	238.9 ± 119.6	251.0 (204.2,295.0)	0.444
Cr (μmoI/L)	64.0 ± 13.6	63.3 (53.0,78.0)	0.832
Na (mmoI/L)	135.0 (135.0,135.0)	135.0 (135.0,135.0)	0.690
PTA (%)	38.8 ± 9.6	26.4 ± 8.1	0.003
INR	2.0 (1.8,2.3)	3.1 ± 1.3	0.003
logHBVDNA log (IU/mL)	5.0 ± 1.5	6.9 (3.5,7.3)	0.158

*CHB, chronic hepatitis B; ACLF, acute-on-chronic liver failure; WBC, white blood cell; LC, lymphocyte count; NC, neutrophil count; NLR, neutrophil-lymphocytes ratio; PLT, platelet; ALT, alanine aminotransferase; AST, aspartate aminotransferase; ALB, albumin; GLO, globulin; TBIL, total bilirubin; Cr, creatinine; Na, sodium; PTA, prothrombin activity; INR, international normalized ratio; p values were calculated by χ^2^ or Fisher exact test for categorical variables and unpaired t test or Mann-Whitney test for continuous variables*.

### Peripheral Frequencies of T Cell Subsets in Patients With HBV-ACLF

**Figure 1:**
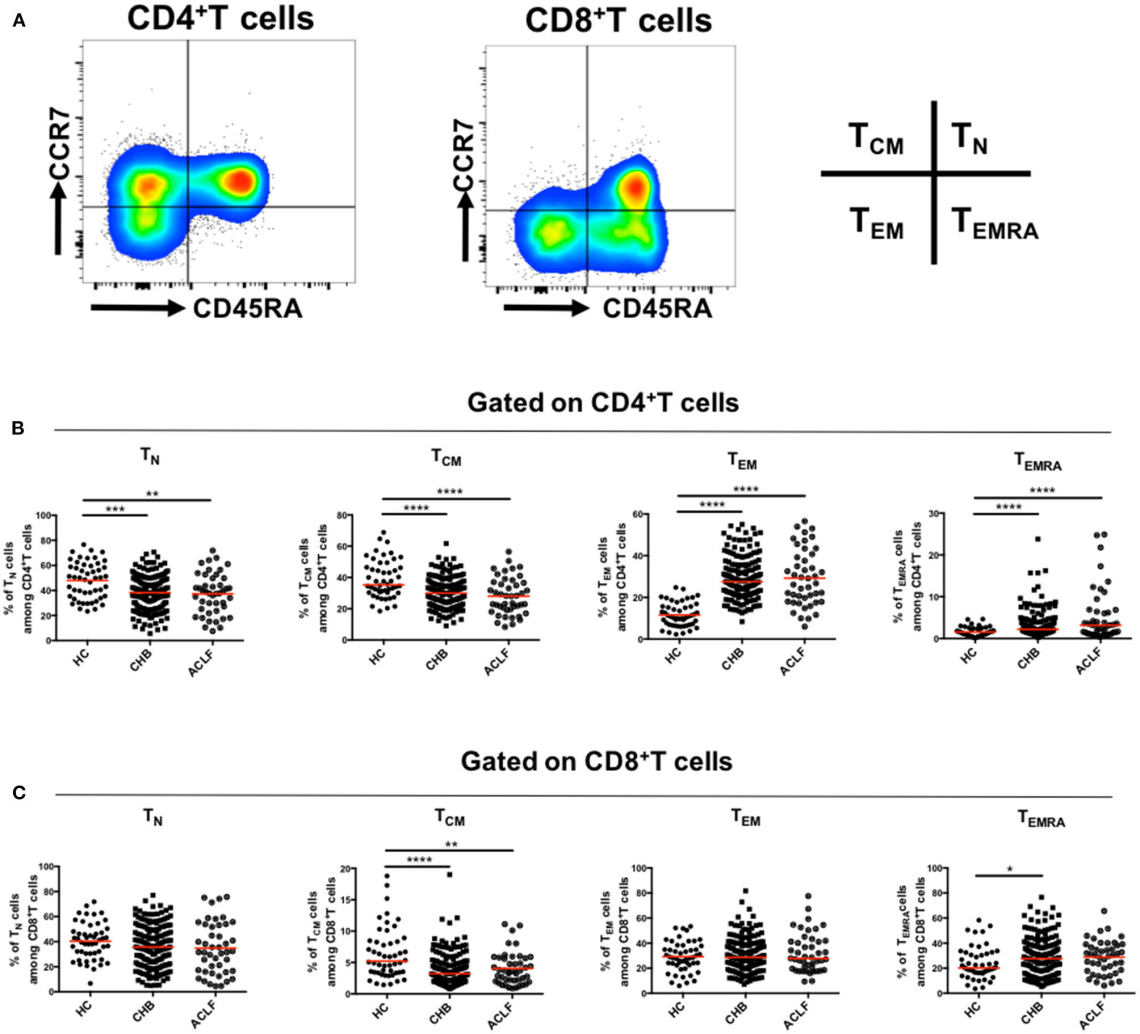
Differentiation of circulating T cells. Representative flow data gated on CD4^+^ and CD8^+^ T cells **(A)** and scatter dot plots of the percentage of T_N_, T_CM_, T_EM_, and T_EMRA_ subsets gated on CD4^+^ **(B)** and CD8^+^T cells **(C)** in different groups. A one-way ANOVA or Kruskal–Wallis test was used to analyze statistical differences. *p < 0.05, **p < 0.01, ***p < 0.001, ****p < 0.0001.

First, we investigated the differentiation status of peripheral T cells in patients with HBV-ACLF, including naïve T cells (T_N_, CCR7^+^CD45RA^+^), central memory T cells (T_CM_, CCR7^+^CD45RA^−^), effector memory T cells (T_EM_, CCR7^−^CD45RA^−^), and terminally differentiated effector cells (T_EMRA_, CCR7^−^CD45RA^+^) (Figure 1A). As shown in Figure 1B, in CD4^+^ T cell subsets, the proportion of T_N_ and T_CM_ cells in patients with HBV-ACLF was significantly lower than that in the HC group, and the proportion of T_EMRA_ cells was significantly higher than that in the HC group. Similar results were observed in CD8^+^ T_CM_ cells and CD8^+^ T_EMRA_ cells (Figure 1C). However, the number of CD8^+^ T_EM_ cells did not increase as in CD4^+^T cells. Additionally, no differences were observed between patients with HBV-ACLF and CHB in these subsets.

**Figure 2:**
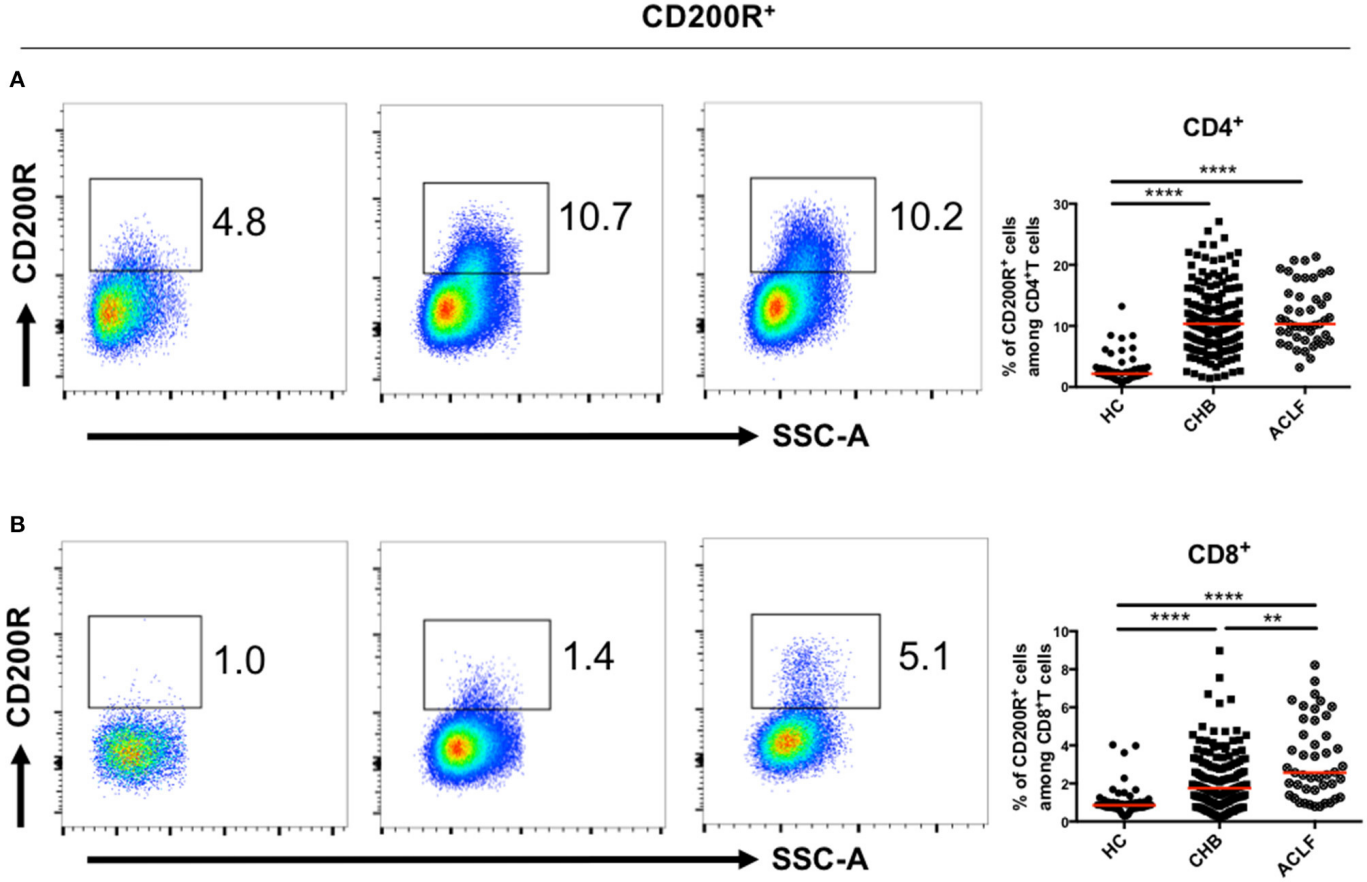
CD200R^+^T cells in patients with ACLF and CHB. Representative dot plots of CD200R^+^CD4^+^ **(A)** and CD200R^+^CD8^+^ **(B)** T cell frequency for each group. A Kruskal–Wallis test was used to analyze statistical differences. **p < 0.01, ****p < 0.0001.

The proportions of CD200R^+^T cells in both CD4^+^ (Figure 2A) and CD8^+^ (Figure 2B) T cells were increased in patients with HBV-ACLF and CHB compared to the HC group. Moreover, the proportion of CD200R^+^CD8^+^T cells in patients with HBV-ACLF was significantly higher than that in patients with CHB.

### CD200R Expression Levels on Different Subsets of Circulating T Cells

**Figure 3:**
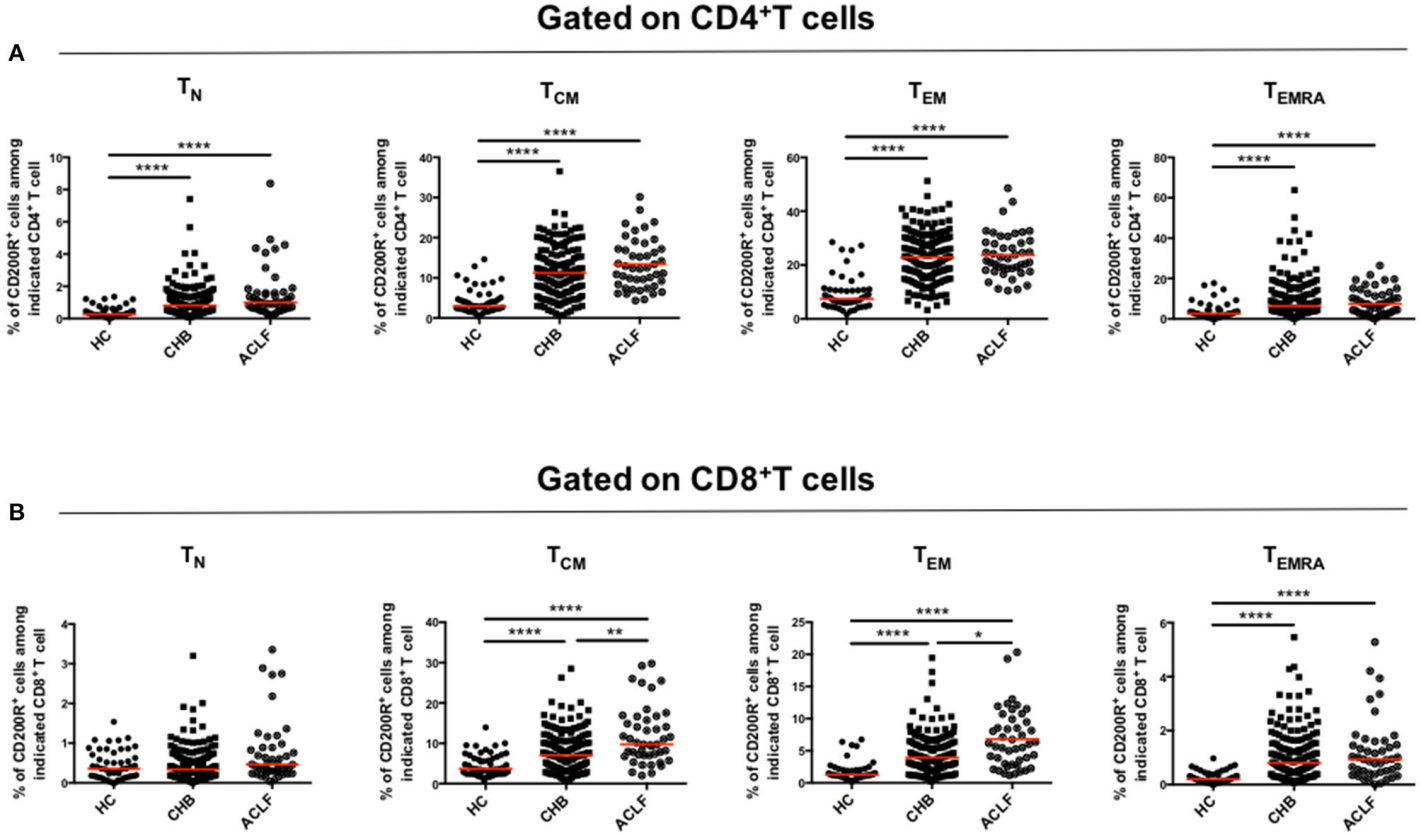
CD200R is significantly upregulated on T_CM_ and T_EM_ cells in patients with ACLF. Scatter dot plots of CD200R expression in each group among four subsets (T_N_, T_CM_, T_EM_, and T_EMRA_) gated on CD4^+^T cells **(A)** and CD8^+^T cells **(B)**. A one-way ANOVA or Kruskal–Wallis test was used to analyze statistical differences. **p < 0.01, ****p < 0.0001.

To further determine the upregulation of CD200R in patients with HBV-ACLF, we investigated whether CD200R was differentially expressed on each differentiation subset of T cells. As shown in Figure 3A, CD200R expression on each differentiation subset was dramatically increased in patients with HBV-ACLF compared with that in HCs on CD4^+^ T cells, along with a more significant increase in the proportions of CD8^+^ T_CM_ and CD8^+^ T_EM_ cells (Figure 3B). This indicated that elevated CD200R^+^ frequency was caused not only by variation in the proportion of T cell subsets, but also the expression on each differentiation subset.

### Decreased Frequency of Circulating CD200R^+^CD4^+^T Cells Was Associated With a Poor Survival Rate for HBV-ACLF

**Figure 4:**
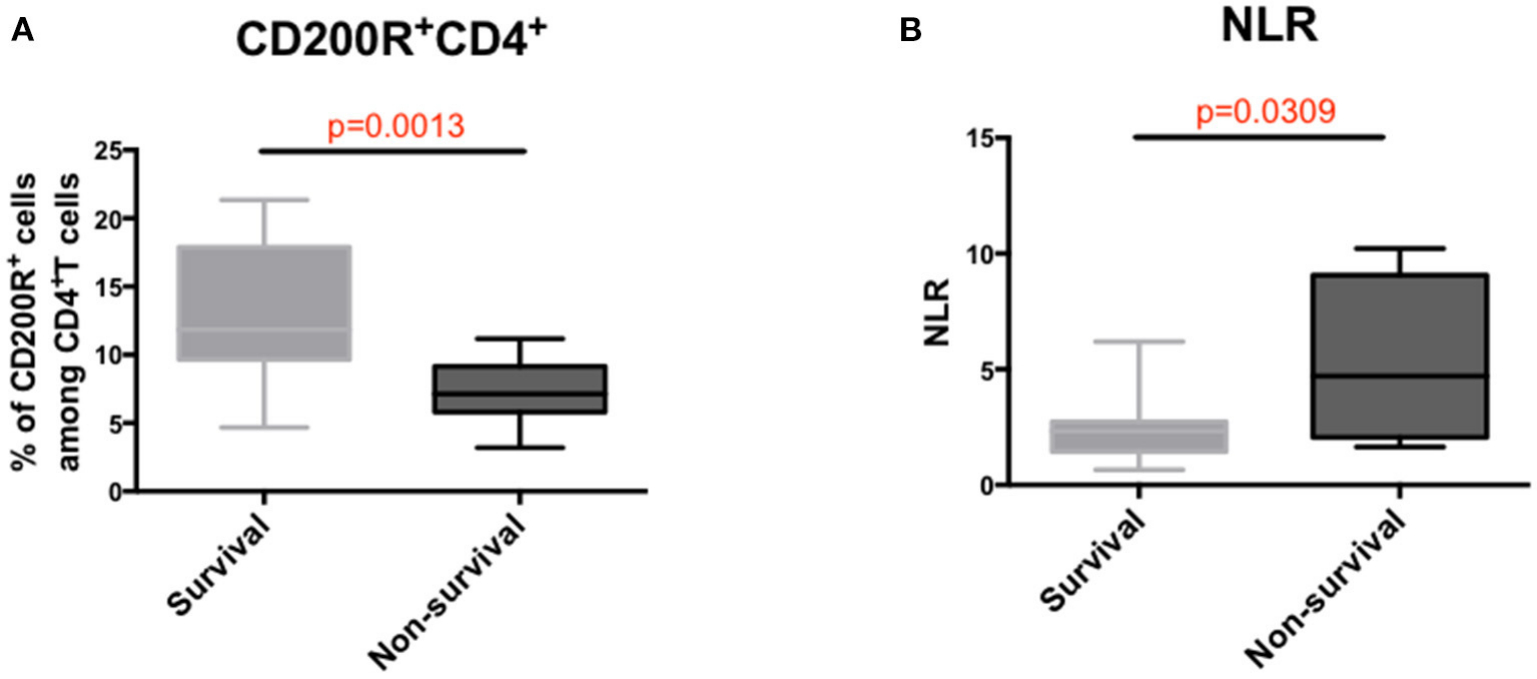
Analysis of prognostic factors in patients with ACLF. **(A)** Comparison of the proportions of CD200R^+^CD4^+^T cells in ACLF survival and non-survival groups. **(B)** Comparison of NLR levels between survival and non-survival groups with ACLF. A Mann–Whitney test was used to analyze statistical differences.

To further investigate the correlation between the indicators and prognosis of HBV-ACLF, we compared the frequencies of the subsets in each group. A lower percentage of CD200R^+^CD4^+^T cells was observed in the non-survival group than in the survival group (*p* = 0.0013) (Figure 4A). Consistent with the findings of our previous study, the NLR was significantly higher in the non-survival group than in the survival group (*p* = 0.0309) (Figure 4B). Additionally, the proportion of CD4^+^ and CD8^+^ T_N_ cells increased significantly in the non-survival group, accompanied by a decreased proportion of CD4^+^ and CD8^+^ T_EM_ subsets (Supplementary Figure 1). Thus, it is suggested that the dysfunction of T_EM_ subsets may not only play a role in the pathogenesis of ACLF but also affect adverse outcomes of ACLF.

### CD200R Combined With the NLR Could Predict HBV-ACLF Prognosis

**Figure 5:**
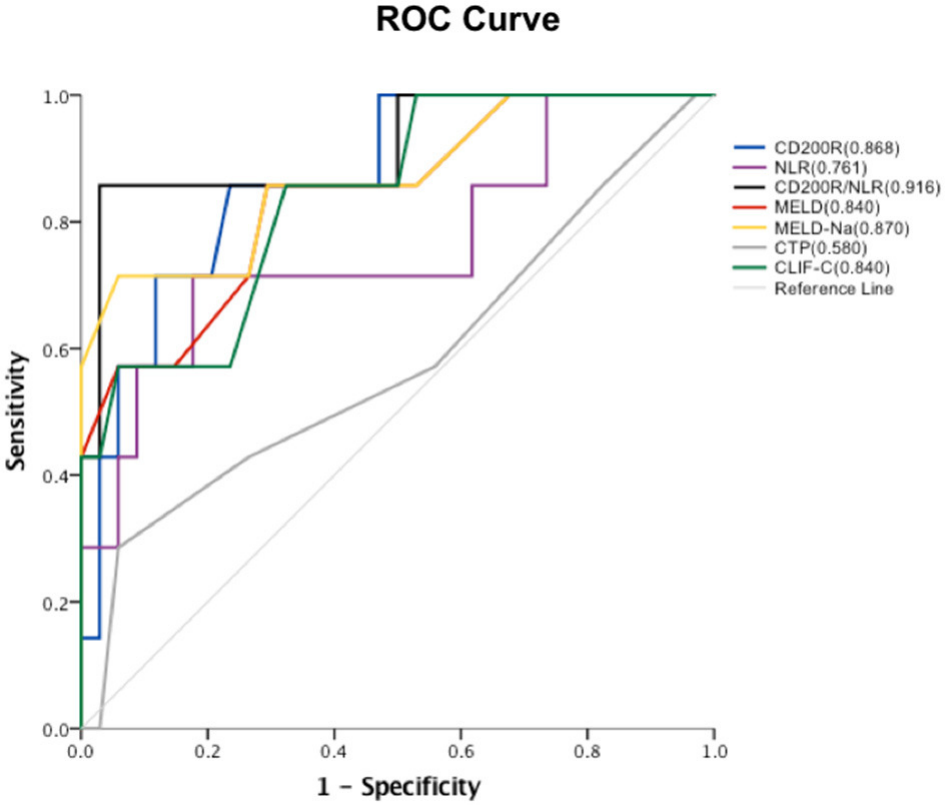
ROC curve of each factor and areas under ROC curves. Proportions of baseline CD200R^+^CD4^+^T cells, NLR, CD200R/NLR, MELD, MELD-Na, CTP and CLIF-C scores in predicting 90 d mortality in patients with HBV-ACLF.

The relationship between baseline CD200R^+^CD4^+^T cell frequency and prognosis for patients with HBV-ACLF was determined at the 90d follow-up. As shown in Figure 5, the baseline level of CD200R^+^CD4^+^T cells yielded an area under the receiver operating characteristic [AUROC (95% CI)] [0.868 (0.733–1.000)] curve that predicted 90 d mortality rate vs. that of NLR, model for end-stage liver disease (MELD), MELD-Na, Child-Turcotte-Pugh (CTP), and chronic liver failure-consortium ACLF (CLIF-C ACLF) score [0.761 (0.538–0.983), 0.840 (0.672–1.000), 0.870 (0.702–1.000), 0.580 (0.322–0.838), and 0.840 (0.684–0.996), respectively]. A combination of CD200R^+^CD4^+^T cells and NLR was used to predict mortality in patients with HBV-ACLF. It was observed that the combination (AUROC [95% CI] [0.916 (0.782–1.000)] predicted 90 d mortality better than that of CD200R^+^CD4^+^T cells alone. At the cut-off point of−3.87, which matched the maximum Youden index determined by ROC analysis, the positive predictive and negative predictive values for mortality were 0.86 and 0.97, respectively.

### Discussion

In the present study, we investigated the frequencies of CD200R^+^ and the differentiation status of T cells in patients with HBV-ACLF and their possible role in predicting prognosis. With chronic HBV infection, the T lymphocyte response plays an important role in host immunity. Elevated expression of coinhibitory receptors on T cells was also found in patients with CHB and acute liver injury and was correlated with disease progression in HBV infection (21, 22). Although increased CD200R expression was found in both CD4^+^ and CD8^+^T cells in patients with HBV-ACLF and CHB, only the percentage of CD200R^+^CD8^+^T cells was further upregulated in patients with HBV-ACLF. Further analysis revealed that upregulation of CD200R occurred in each differentiation subset. Moreover, although the percentage of CD8^+^T_CM_ cells was decreased as that of CD4^+^T_CM_ cells, the level of CD8^+^T_EM_ cells was not upregulated, as in CD4^+^T cells. This suggests that further dysfunction of T cells, especially defective CD8^+^T cell function, may contribute to the pathogenesis of HBV-ACLF.

Interestingly, in contrast to the increase in peripheral frequencies of CD200R^+^T cells in patients with HBV-ACLF and CHB compared with HCs, CD200R^+^CD4^+^T cells in the non-survival HBV-ACLF group were decreased. The 90 d prognosis analysis of patients with HBV-ACLF but without liver transplantation showed that the proportion of CD200R^+^CD4^+^T cells in the HBV-ACLF non-survival group was lower than that in the survival group. Notably, the opposite tendencies were observed in T_N_ cells, demonstrating statistical differences between the survival and non-survival groups. Compared with the healthy control group, the proportion of CD4^+^T_N_ cells in patients with HBV-ACLF decreased and differentiated into effector T cells, whereas the proportion of effector memory CD8^+^T cells did not change significantly. In summary, the above results suggest that T cells might play different roles in the pathogenesis and prognosis of HBV-ACLF.

Previous studies have found that CD200 and CD200R can provide negative regulatory signals, change the response threshold of myeloid cells to stimulation signals, reduce the activity of myeloid cells, and maintain immune homeostasis (23–25). Multiple effects on T cells have been reported in interactions between CD200/CD200R, including the shift from a Th1 cytokine profile to a Th2 cytokine profile (26) and the inhibition of the CTL response (27). Ren Y et al. (28) revealed that the CD200/CD200R interaction could reduce the differentiation of CD4^+^T cells into Th17 cells in rheumatoid arthritis, downregulate Th17 chemotaxis mediated by chemokine receptor 6, and reduce the inflammatory response. Additionally, the CD200/CD200R interaction could indirectly regulate T cell function through macrophages or dendritic cells (29, 30). Therefore, the decrease in the proportion of CD200R^+^CD4^+^T cells could possibly promote the inflammatory response and aggravate tissue damage, affecting the prognosis of patients with HBV-ACLF. To further verify this hypothesis, additional functional experiments must be conducted, and the identification of the interaction between CD200R and other coinhibitory molecules should be the focus of future studies.

Our study findings also showed that the baseline percentage of CD200R^+^CD4^+^T cells was a potential predictive marker for 90 d mortality in patients with HBV-ACLF. NLR has been proved to be a predictor of the prognosis in patients with HBV-ACLF (18). A combination of CD200R and NLR provided a better prediction of 90 d mortality than CD200R alone. This further illustrates the potential importance of CD200R in the prognosis of HBV-ACLF.

In summary, during the development of HBV-ACLF, the role of T cells in promoting or suppressing inflammation may shift with variations in regulatory factors. CD200R, as a potential predictor and possible mechanism of HBV-ACLF pathogenesis, is worthy of further study. However, there were some limitations to our study. The number of cases was limited, and subjects were recruited from a single center; thus, further validation and mechanistic research need to be performed in the future.

## Conclusion

Overall, CD200R combined NLR offers potential predictive value regarding the mortality of HBV-ACLF, and the findings could contribute to the elucidation of the pathogenesis of HBV-ACLF.

## Data Availability Statement

The raw data supporting the conclusions of this article will be made available by the authors, without undue reservation.

## Ethics Statement

The studies involving human participants were reviewed and approved by Ethics Committee of the Beijing Ditan Hospital, Capital Medical University. The patients/participants provided their written informed consent to participate in this study.

## Author Contributions

XW and HZ were responsible for the conception, design of the study, revised, and commented on the draft. YL and YK performed the analysis and interpretation of the data. KS, YH, QZ, and BZ participated in the data collection and follow-up of patients. YL drafted the manuscript. All authors contributed to the article and approved the submitted version.

## Funding

This research was supported by National Natural Science Foundation of China (81774234 and 81804009) and Beijing Municipal Administration of Hospitals Clinical Medicine Development of Special Funding Support (ZYLX201707).

## Conflict of Interest

The authors declare that the research was conducted in the absence of any commercial or financial relationships that could be construed as a potential conflict of interest.

## Publisher's Note

All claims expressed in this article are solely those of the authors and do not necessarily represent those of their affiliated organizations, or those of the publisher, the editors and the reviewers. Any product that may be evaluated in this article, or claim that may be made by its manufacturer, is not guaranteed or endorsed by the publisher.

## Supplementary Material

Supplementary Figure 1 Analysis of prognostic factors in patients with ACLF. Comparison of T cell differentiation between survival and non-survival groups with ACLF. Mann–Whitney or unpaired *t* test was used to analyze statistical differences.
