# Supplementary figures and images for: CD200R Combined Neutrophil-Lymphocyte Ratio Predict 90-Day Mortality in HBV-Related Acute-On-Chronic Liver Failure
Source: Front Med (Lausanne). 2021 Dec 6;8:762296. doi: 10.3389/fmed.2021.762296 (PMC8685236; doi:10.3389/fmed.2021.762296)

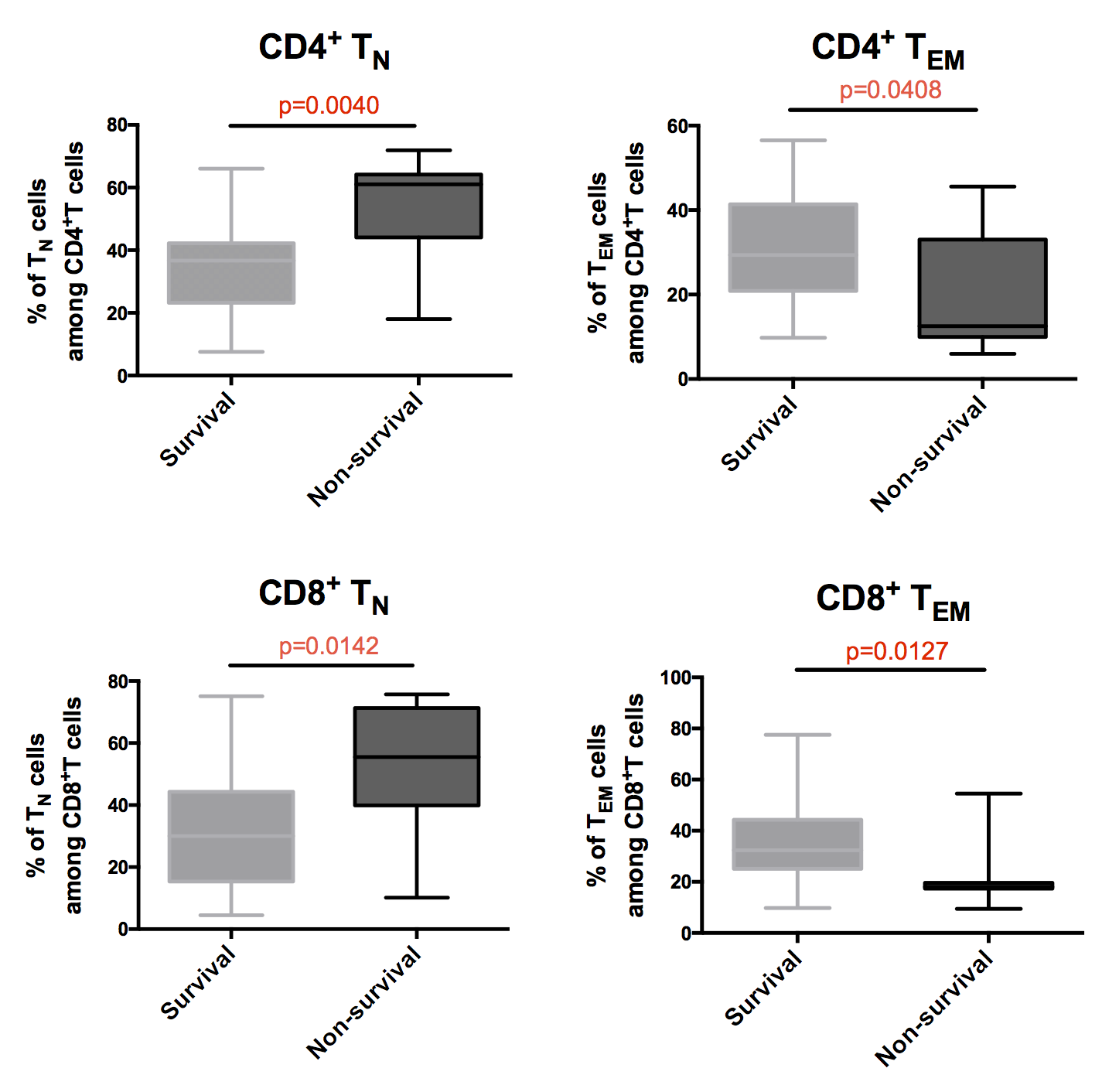

Supplement: Supplementary Figure 1 — Analysis of prognostic factors in patients with ACLF. Comparison of T cell differentiation between survival and non-survival groups with ACLF. Mann–Whitney or unpaired t test was used to analyze statistical differences. [file Image_1.TIFF]
